# The effect of neuro-enhancement technology on proprioception in patients with anterior cruciate ligament reconstruction

**DOI:** 10.3389/fnhum.2025.1651309

**Published:** 2025-09-15

**Authors:** Hongzhen Du, Xipeng Wu, Hao Zhang, Yuxuan Zhang, Zihao Sun, Baobin Mi, Wei Li

**Affiliations:** ^1^School of Special Education and Rehabilitation, Binzhou Medical University, Yantai, Shandong, China; ^2^Department of Rehabilitation Medicine, Binzhou Medical University Hospital, Binzhou, Shandong, China; ^3^Department of Rehabilitation, Laizhou People's Hospital, Yantai, Shandong, China; ^4^Department of Critical Care Rehabilitation II, Shandong Provincial Third Hospital, Jinan, Shandong, China; ^5^Department of Geriatrics, Binzhou Medical University Hospital, Binzhou, Shandong, China

**Keywords:** anterior cruciate ligament reconstruction, proprioception, repetitive transcranial magnetic stimulation, balance control, joint position sense

## Abstract

**Objective:**

To investigate whether the neural augmentation technique can induce improvement of proprioceptive performance in Anterior cruciate ligament reconstruction (ACLR) patients.

**Methods:**

Forty ACLR patients were recruited and randomly assigned to receive either active prefrontal cortex-targeted repetitive transcranial magnetic stimulation (rTMS) or sham stimulation (20 cases per group). All participants underwent the allocated intervention (active or sham rTMS over the prefrontal cortex) and completed standardized balance and proprioceptive assessments both pre- and post-intervention to evaluate the neuromodulatory effects on proprioceptive function in ACLR patients. The alpha level for statistical significance was set at *ρ* ≤ 0.05 *a priori*.

**Results:**

In the rTMS group, the Center of Pressure Area (COPA) and the Center of Pressure Sway (COPS) of the injured limb were significantly reduced before and after repeated transcranial magnetic stimulation (*p* = 0.002), and the 30° positional sensory stimulation was significantly improved compared with the pre-stimulation period (*p* = 0.012).

**Conclusion:**

Neuro-enhancement technology can improve the proprioceptive performance of ACLR patients and thus improve their motor ability.

## 1 Introduction

Anterior cruciate ligament (ACL) injuries represent a prevalent category of knee ligament trauma in sports medicine, particularly among athletes, significantly impairing motor function and occupational capacity in young adults (Konishi, 2018). Anterior cruciate ligament reconstruction (ACLR) is widely regarded as the gold-standard treatment, aiming to restore knee joint biomechanical stability and facilitate a return to pre-injury activity levels (Hughes et al., 2020). However, clinical observations reveal suboptimal postoperative functional recovery rates, with at least 20% of patients failing to regain pre-injury motor performance. Beyond mechanical instability, pain, kinesiophobia, and arthrogenic muscle inhibition, emerging evidence implicates proprioceptive dysfunction as a critical contributing factor (Kaya et al., 2019).

Proprioception—a cornerstone of sensorimotor integration—enables spatial perception of body position and coordinated movement execution (Moon et al., 2021). The ACL contributes crucially to proprioceptive feedback during dynamic activities, modulating knee stability and neuromuscular control (Xu et al., 2022). This feedback mechanism is essential for balance maintenance, dynamic motion control, and injury prevention, especially in athletic populations. ACLR patients frequently exhibit proprioceptive deficits due to the injury’s pathoanatomy, surgical intervention, and rehabilitation processes (Arumugam et al., 2021), which may precipitate functional impairment and elevated reinjury risk (Forelli et al., 2023). Post-injury and reconstruction, associated ligamentous structures and neural pathways often remain compromised, with deficits persisting beyond structural healing (Zandiyeh et al., 2019). Therefore, comprehensive rehabilitation must address not only mechanical joint restoration but also the crucial re-establishment of proprioceptive acuity (Chaput et al., 2022).

Joint position sense (JPS) is the most commonly used method to assess proprioceptive deficits, typically involving passive or active reproduction of joint angles under visual occlusion (Hillier et al., 2015). ACLR patients exhibit impaired JPS accuracy (Zhao et al., 2023). Center of pressure Area (COPA) refers to the envelope area of the center of pressure trajectory, reflecting overall static balance stability, while center of pressure Sway (COPS) measures the displacement amplitude of the center of pressure in the anterior–posterior and medial-lateral directions, quantifying postural sway. Both are closely related to lower-limb proprioception—declines in proprioceptive accuracy lead to increased COPA area and greater COPS sway, indicating impaired balance control (Horváth et al., 2023).

Concurrently with proprioceptive deficits, ACLR patients develop neuroplastic adaptations in the prefrontal cortex (Grooms et al., 2017). Compared to healthy controls, ACLR patients demonstrate significantly reduced prefrontal activation during motor tasks, with the degree of proprioceptive deficit showing positive correlation with prefrontal engagement (Cao et al., 2024; Strong et al., 2022). This neural divergence suggests impaired recruitment of prefrontal resources to support complex motor tasks. To maintain post-injury dynamic knee stability (Gokeler et al., 2019), ACLR patients exhibit heightened cognitive and cross-modal neural activity for basic knee motor control (Chaput et al., 2024). Such neural compensation may foster excessive reliance on visual-cognitive processing to sustain fundamental functions like proprioception and dynamic stability (Miko et al., 2021), shifting sensorimotor strategies toward visuomotor dependence.

Proprioception-focused training demonstrates improved intervention outcomes when combined with central neuromodulation (Liu et al., 2024). Among these approaches, repetitive transcranial magnetic stimulation (rTMS) has emerged as a promising adjunct (Veldema and Gharabaghi, 2022). As a non-invasive brain stimulation technique, rTMS utilizes pulsed magnetic fields to modulate cortical excitability, enabling targeted neuromodulation (Klomjai et al., 2015). Its therapeutic efficacy has been established in stroke rehabilitation, chronic pain management, and neurodegenerative diseases (Lefaucheur et al., 2020). Notably, rTMS exhibits neurophysiological potential for enhancing proprioceptive function and facilitating sensorimotor recovery (Poh et al., 2022). The mechanism of rTMS-mediated proprioceptive improvement involves synaptic plasticity modulation within the central nervous system. Specifically, rTMS may activate relevant brain regions and induce cortical reorganization, augmenting the brain’s compensatory capacity post-injury (Jannati et al., 2023). For ACLR patients with disrupted proprioceptive pathways, rTMS could potentially restore neural circuit functionality (Xia et al., 2023). By enhancing prefrontal cortical excitability and functional connectivity, rTMS may optimize proprioceptive input processing efficiency, thereby ameliorating visuomotor dependence and improving rehabilitation responsiveness (Zheng et al., 2024). rTMS improves vestibular dependent balance control by targeting the cerebellum and optimizes motor planning and posture control for balance tasks by acting on brain regions related to the prefrontal lobe (Parikh et al., 2024; Schoeberl et al., 2024). Nevertheless, existing research on rTMS for proprioceptive enhancement in musculoskeletal disorders—particularly ACLR—remains limited. Prior studies predominantly focus on balance and gait tasks in stroke or multiple sclerosis populations (Hofmeijer et al., 2023), findings that may not directly translate to ACLR’s unique sensorimotor requirements. Consequently, systematic investigation into rTMS’s specific effects on proprioceptive performance in ACLR patients is imperative.

This study employs a randomized sham-controlled design to evaluate rTMS-induced improvements in proprioceptive performance among ACLR patients, as measured by trajectory length reduction and stability enhancement during balance tasks, along with improved joint position sense testing outcomes. We hypothesize that active rTMS will significantly enhance proprioceptive performance compared to sham stimulation. By elucidating the role of rTMS-based neuro-enhancement technology in ACLR proprioceptive recovery, this study advances discussions on innovative rehabilitative interventions through dual physiological and functional perspectives. The findings will establish a foundation for subsequent research while deepening understanding of neurophysiological mechanisms underlying post-injury proprioceptive rehabilitation.

## 2 Methods

### 2.1 General information

Forty patients who underwent ACLR in the Binzhou Medical University Hospital from January 2024 to October 2024 were selected and randomly divided into rTMS group and sham group. After confirming normality with the Shapiro–Wilk test, continuous baseline characteristics were compared using paired t-tests. Comparison of demographic and clinical characteristics between groups showed no statistically significant differences (*p* > 0. 05) (Table 1). All participants provided written informed consent prior to enrollment. This study was approved by the Ethics Committee of the Affiliated Hospital of Binzhou Medical University (under the Ethical Approval Number 2022-G 29–01).

**Table 1:** Classification of characteristics.

Variable	rTMS	Sham
*N*	20	20
Age (years)	27.10 ± 4.41	28.10 ± 4.71
Height (cm)	169.80 ± 8.38	170.85 ± 7.67
Weight (kg)	66.4 ± 10.60	64.2 ± 10.81
Body Mass Index (kg/m^2^)	22.99 ± 3.01	21.85 ± 2.12

### 2.2 Inclusion criteria

The inclusion criteria were as follows: (1) MRI showing simple ACL injury with good ligament tissue structure; (2) no meniscal injury; (3) no presence of internal tumors, infection, fracture; (4) no mental illness; (5) no unstable vital signs in major organs such as the heart, brain, and kidney; (6) no ACL injury secondary to immune and metabolic diseases; (7) no severe osteoporosis; (8) no venous thrombosis; (9) not pregnant or lactating women; (10) informed consent and voluntary cooperation of patients; (11) unilateral knee injury; (12) absence of periarticular muscle atrophy (Palke et al., 2022); (13) no limitation in knee joint range of motion (Proske, 2019).

### 2.3 Test program

**Figure 1:**
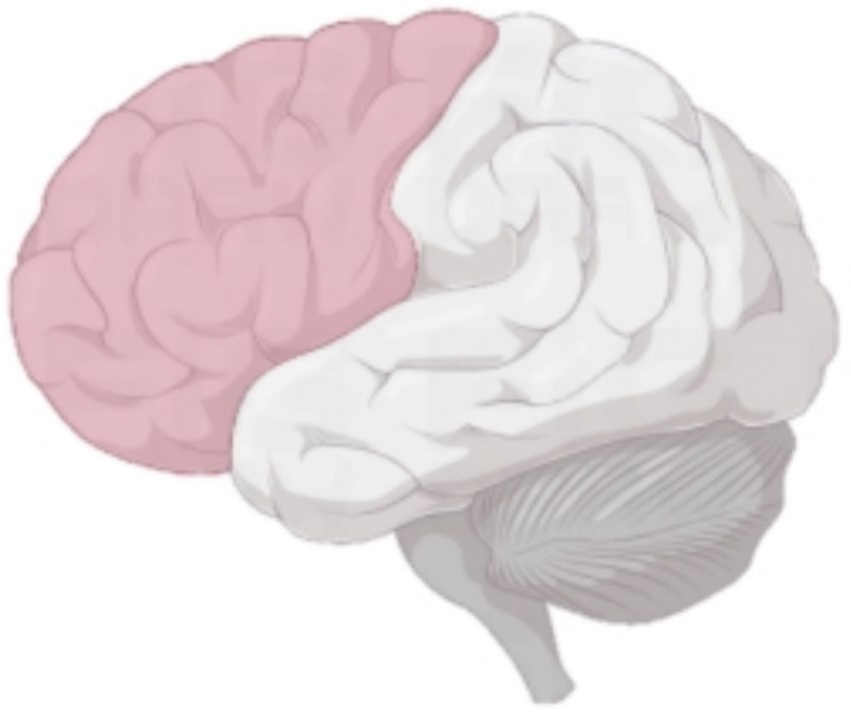
The prefrontal region of the cerebral cortex.

Prior to stimulation, all participants completed a balance test and a proprioceptive test. rTMS was applied to the patients in the experimental group using standard treatment parameters (Choi et al., 2016; Kakuda et al., 2013), with the stimulation intensity set at 10% subthreshold to the resting motor threshold, a frequency of 10 times per second, and each stimulation pulse lasting 5 s at 25-s intervals for a period of 20 min. In the sham group, the coils were rotated 90°on their handles to position them tangentially to the scalp. While this adjustment allowed patients to hear the instrument’s clicking sound during treatment, it effectively prevented the delivery of actual neural stimulation. The stimulation target was the unilateral prefrontal cortex contralateral to the injured limb, based on the principle of central nervous system cross-lateral control (Figure 1). After stimulation, participants were again administered a balance test and a proprioceptive test to assess the effects of repetitive rTMS on proprioception in ACLR patients. The exclusion criteria were as follows: (1) Limited joint movement prevents accurate testing; (2) patients unable to complete evaluation and rehabilitation training as required. None of the subjects reported any adverse side effects concerning pain on the scalp or headaches after the experiment.

### 2.4 Plantar center of pressure (COP) assessment

All subjects were assessed for postural stability in the standing position using the Fourier Intelligence Balance Function Training Assessment System (model AL-600; Fourier Intelligence, Shanghai Fourier Intelligence Technology Co., Ltd., Shanghai, China) (Liu et al., 2016). The system consists of a pressure test plate and a computerized system. The middle part of the pressure test plate is equipped with pressure sensors, which receive vertical pressure changes from the feet and analyze the COP changes, reflecting the COP position and movement; the computer system runs the PelmaMotus software, which analyzes the incoming data from the force plate and records the changes in COP position during the test.

Before each use, the pressure test board was firstly corrected, and after correction, standing on both feet in the fixed position of the pressure test board, with arms hanging down, eyes looking straight ahead, keeping steady, the frame rate of the test board was 100 frames/s. The evaluation indexes are as follows: ① COPA: COPA is the area of the COP envelope graph of each frame, reflecting the limit range of the human body’s movement of the center of pressure, and when the range of movement is smaller, the smaller the value; ② COPS: COPS is the ratio of the length of the COP trajectory to the test time, reflecting the human body’s micro-postural control function. The test time is fixed, when the COP trajectory is shorter, the smaller this value is.

### 2.5 Knee joint position perception measurement

Joint angle error testing is a viable method to assess clinical joint proprioception, which can be used to accurately determine the position of a specific body part in space by measuring the degree of angular deviation from the starting position. Our study assessed the proprioceptive state using isokinetic dynamometer NX A8-3 (Yikang, Guangzhou, China). The outcome variables were measured in the following four trials: (1) measurement of injured limb 30°; (2) measurement of uninjured limb 30°; (3) measurement of injured limb 60° and (4) measurement of uninjured limb 60°. We have been trained as orthopedic rehabilitation therapists prior to the start of the trial, and after achieving a uniform standard, two senior physicians from the rehabilitation assessment team conducted a position perception assessment.

The specific operations were as follows: The knee was moved from a 90° flexion starting position passively to each of the target angles of 30° and 60°. We have reminded patients to close their eyes before collecting data. Please patient hold the leg in the 30° knee extension and 60° knee extension positions for 10s to allow the patient to memorize the position, and was then returned to 90° knee flexion. After a pause of 10 s, the patient, with the memory of the active knee flexion, moves the lower limb in the same way by active contractions and stops when the patient perceives that the target angle has been reached. The mean values of the six trials were obtained for each patient at each angle and used to calculate the difference between the actual angle achieved and the target angle. The smaller the difference, the better the patient’s perception of the position.

### 2.6 Statistical methods

All statistical analyses were performed using Statistical Package for the Social Sciences (SPSS) software (version26.0, IL, USA). The Shapiro–Wilk test was applied to test the normality of all data. However, all data failed the normality tests. Due to non-normality of the data, Wilcoxon signed-rank tests were performed for related samples comparisons. Differences were considered statistically significant at *p* < 0.05. Data plots were obtained using GraphPad Prism 8 software.

## 3 Results

**Figure 2:**
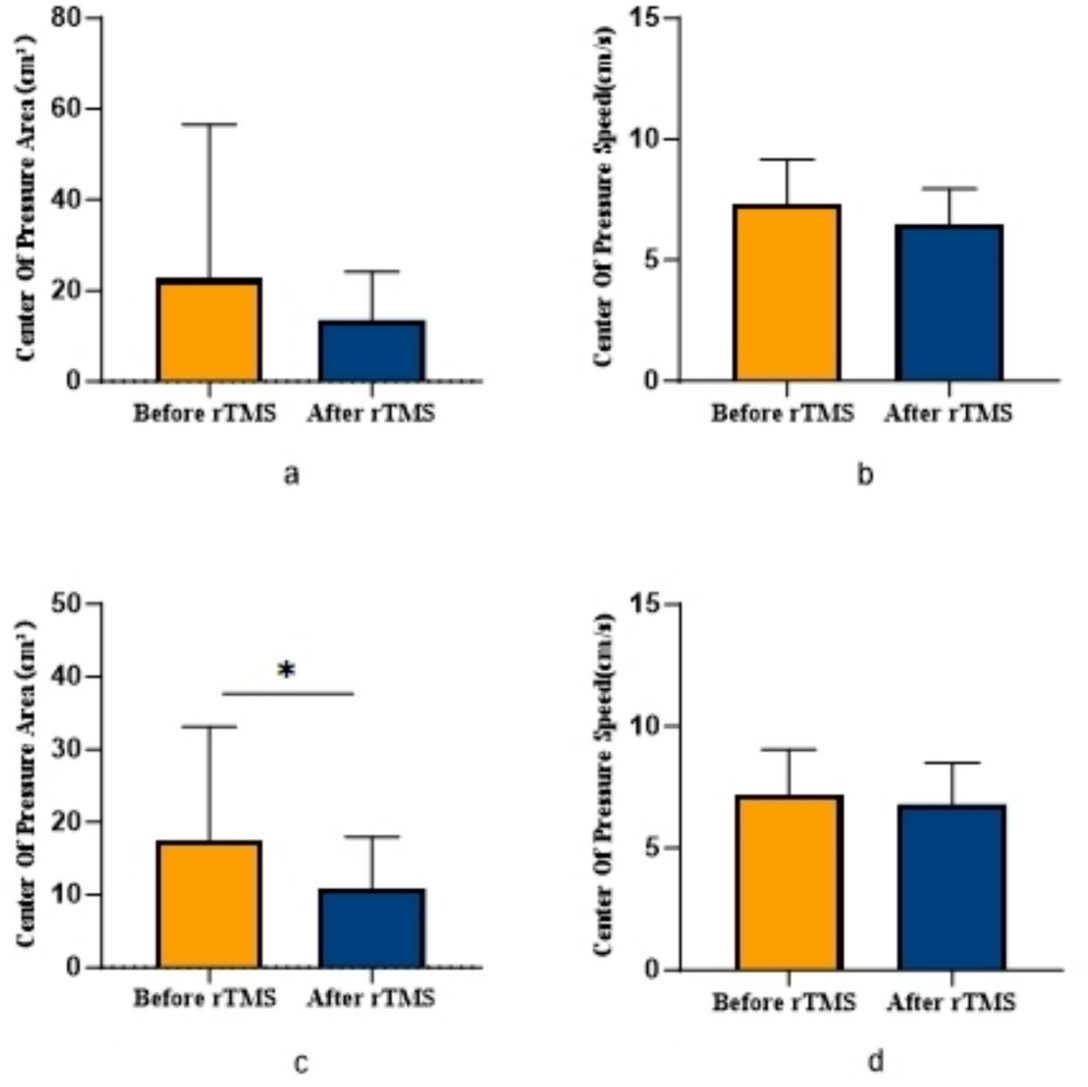
Comparison of COPA and COPS in patients with ACLR in rTMS group. *p < 0.05. **(a)** COPA in the uninjured limb, **(b)** COPS in the uninjured limb, **(c)** COPA in the injured limb, **(d)** COPS in the injured limb.

**Figure 3:**
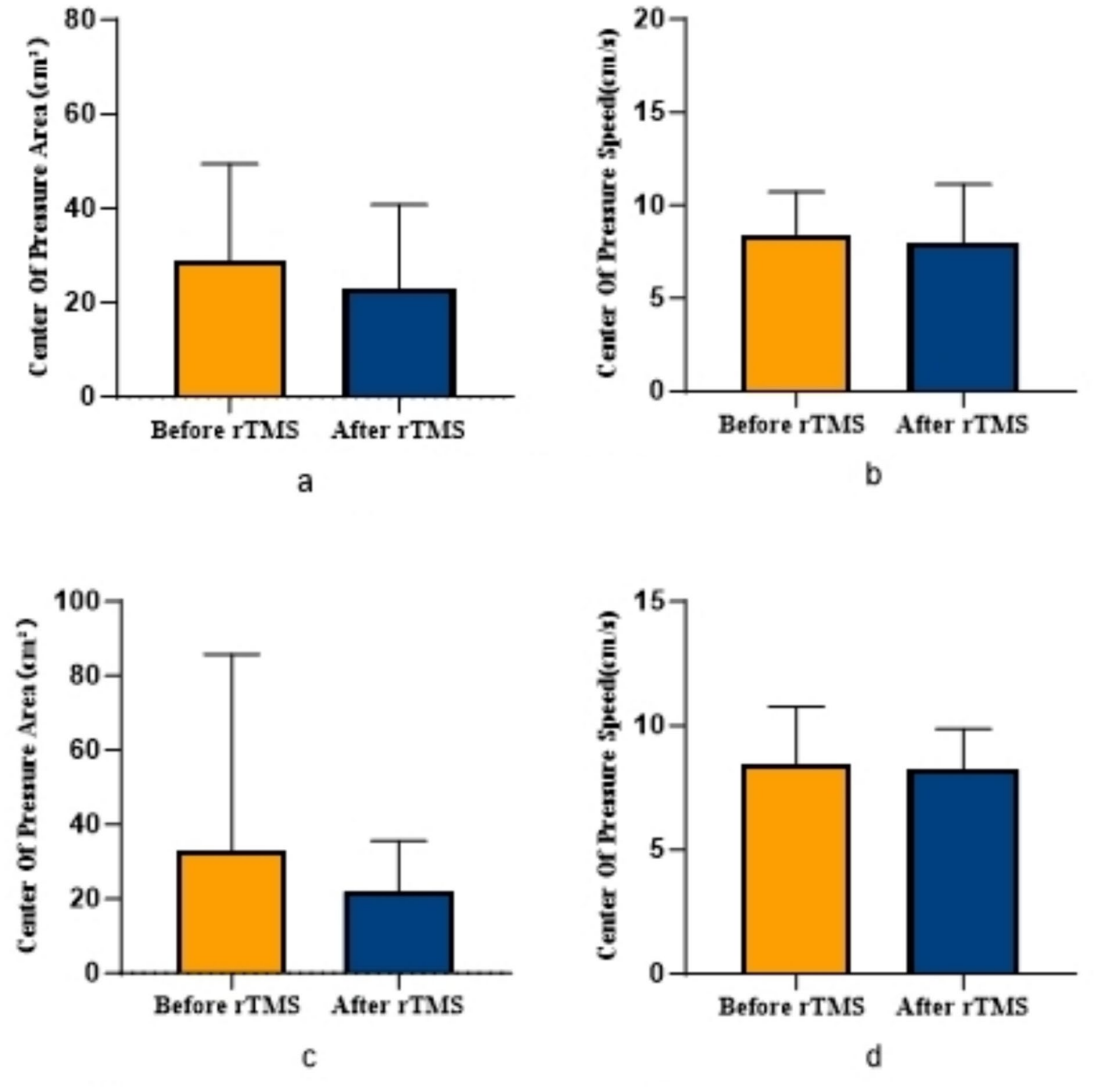
Comparison of COPA and COPS in patients with ACLR in sham group. **(a)** COPA in the uninjured limb, **(b)** COPS in the uninjured limb, **(c)** COPA in the injured limb, **(d)** COPS in the injured limb.

**Figure 4:**
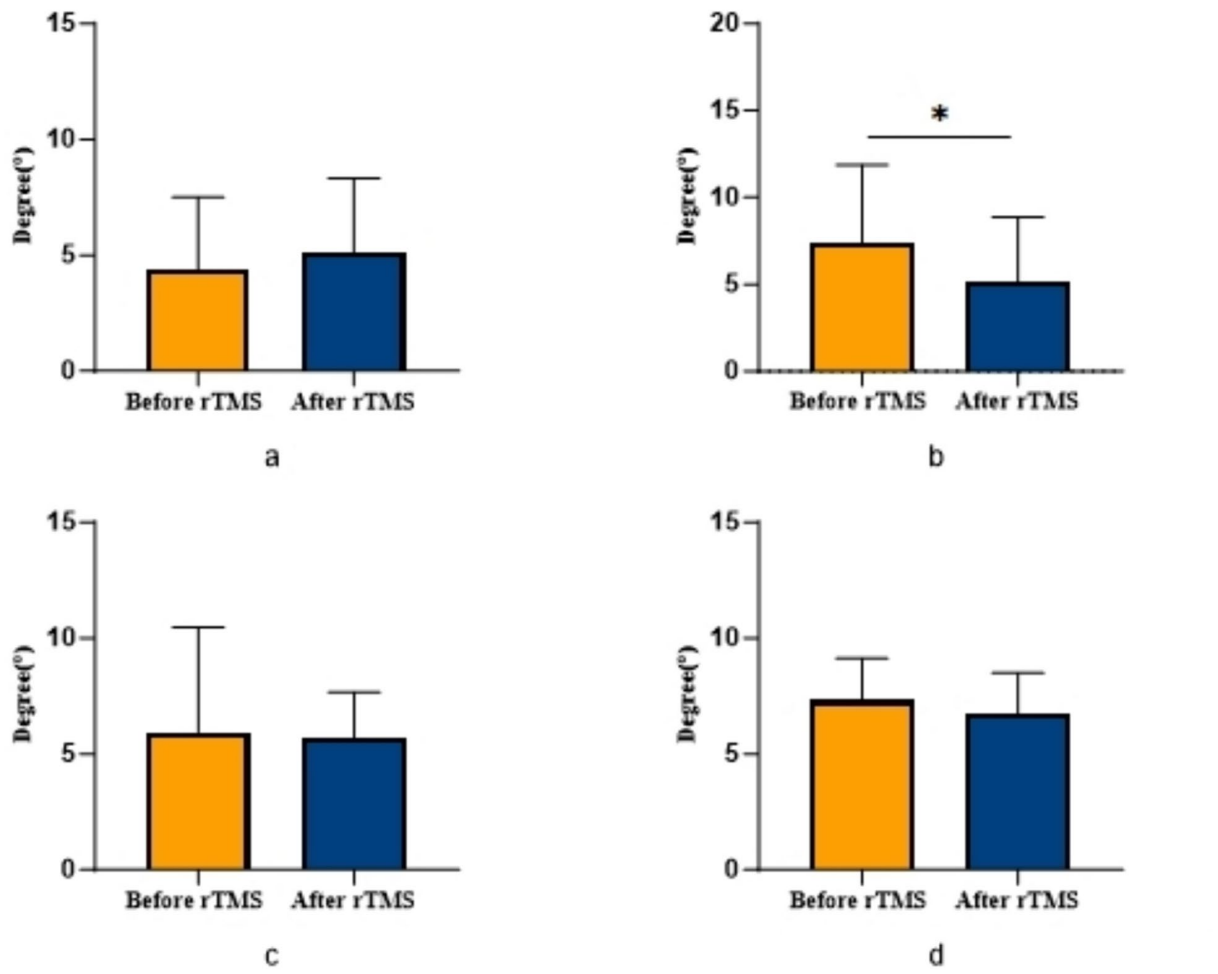
Comparison of bilateral lower limb position perception in patients with ACLR in rTMS group. *p < 0.05. **(a)** The position sense of 30° in the uninjured limb, **(b)** The position sense of 30° in the injured limb, **(c)** The position sense of 60° in the uninjured limb, **(d)** The position sense of 60° in the injured limb.

**Figure 5:**
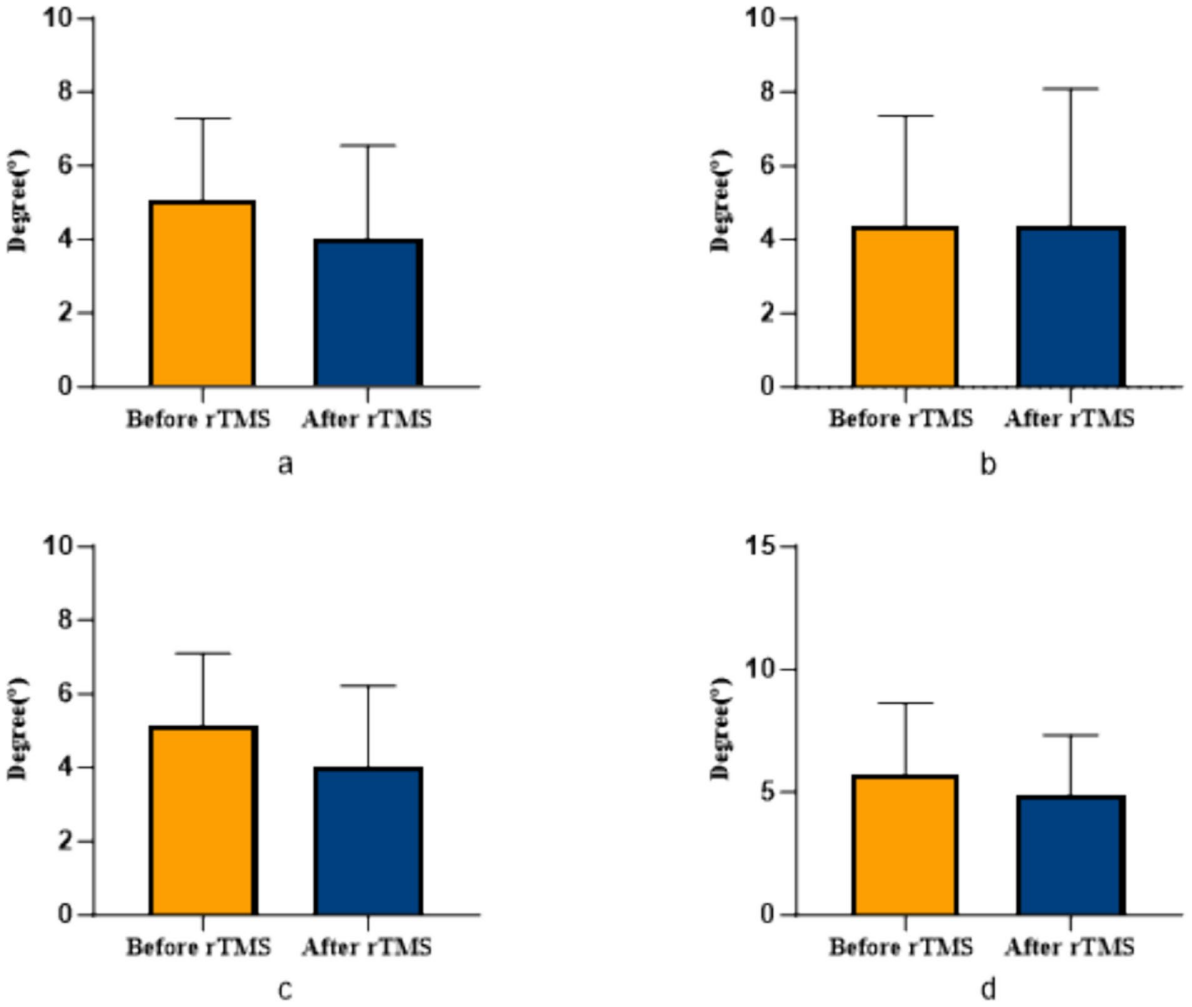
Comparison of bilateral lower limb position perception in patients with ACLR in sham group. **(a)** The position sense of 30° in the uninjured limb, **(b)** The position sense of 30° in the injured limb, **(c)** The position sense of 60° in the uninjured limb, **(d)** The position sense of 60° in the injured limb.

COPA on the injured limb of the rTMS group before and after treatment was greater than that after treatment (*p* = 0.002) (Figure 2); there was no statistically significant difference between COPA and COPS on the uninjured limb of the true stimulation group and the uninjured limb of the sham stimulation group before and after treatment (Figure 3). The 30° position perception on the injured limb of the rTMS group waited until after treatment to get better and there was a statistically significant difference between the two sides of the group (*p* = 0.012) (Figure 4); there was no statistically significant difference between the 30° and 60° position perception of the uninjured limb of the rTMS group and the uninjured limb of the sham group in the before-after treatment comparison (Figure 5). There was no statistically significant difference between the 30° and 60°position perception of the both side of the rTMS group and the sham group.

## 4 Discussion

Our findings demonstrate significant improvements in proprioceptive measures following rTMS intervention, specifically evidenced by reduced COPA and COPS during balance tests, with particularly pronounced COPA reduction in the injured limb of the rTMS group. The marked enhancement in 30° joint position sense further confirms rTMS’s efficacy in modulating central proprioceptive mechanisms. In contrast, the sham stimulation group showed no significant proprioceptive changes, reinforcing that observed benefits derive from rTMS-specific intervention rather than temporal effects or participant bias.

Proprioception—the ability to perceive body segment position and movement effort—is critical for balance and coordinated movement in ACLR patients (Ventura et al., 2024). These patients commonly exhibit proprioceptive deficits that cause abnormal peripheral afferent signals, leading to central compensation through enhanced prefrontal cognitive control and somatosensory cortex processing (An et al., 2022). When performing complex tasks, ACLR patients require stronger prefrontal-premotor co-activation to maintain motor control. This neural adaptation may result in insufficient cognitive resources during dual/multi-tasking situations, representing a neuroplastic barrier to functional recovery and a contributor to reinjury risk (Hu et al., 2023).

While neural augmentation techniques have shown benefits for balance and motor tasks across various clinical conditions (Schoeberl et al., 2024; Menezes et al., 2024), their application to proprioceptive recovery remains limited. rTMS has been confirmed to selectively modulate regional cortical excitability. By enhancing prefrontal-premotor circuit excitability, rTMS effectively reduced injured limb COPA and improved 30° joint position sense, demonstrating its capacity to address proprioceptive deficits common in ACLR patients (Gilio et al., 2009).

Our unilateral prefrontal stimulation protocol warrants consideration. Given that intrahemispheric prefrontal connections to motor/sensory regions substantially exceed interhemispheric connections, combined with ACLR patients’ characteristic interhemispheric imbalance (reduced excitability in the hemisphere contralateral to injury with compensatory hyperexcitability ipsilaterally), rTMS appears to restore this equilibrium (An et al., 2022). This explains why statistically significant improvements primarily manifested in the injured limb despite bilateral trend-level enhancements.

Previous studies note that while ACLR patients’ proprioception generally improves over time, persistent deficits remain particularly evident at 30° joint position testing, with minimal differences at 60° (Zhao et al., 2023; Zhang et al., 2018). This discrepancy may stem from differential muscle recruitment: at 60° flexion, greater quadriceps and hamstring activation (including hamstring isometric contraction and quadriceps-hamstring co-contraction) generates substantial muscular afferent input that compensates for ACL-mediated proprioceptive loss, accounting for limited 60° position sense changes in our study.

Our results underscore proprioception’s pivotal role in athletic performance and functional recovery. Addressing proprioceptive deficits through rTMS may ameliorate balance impairments, agility deficits, and neuromuscular control abnormalities—key factors contributing to elevated reinjury risk in ACLR patients (Buckthorpe, 2021). Incorporating rTMS as an adjunct to conventional physiotherapy could potentially accelerate rehabilitation timelines and optimize functional outcomes.

## 5 Limitations

This study has certain limitations, including a relatively small sample size (n = 40). To enhance the robustness and generalizability of the findings, we plan to conduct larger-scale, multicenter trials in the future. Additionally, long-term follow-up studies will be implemented to validate the sustained effects of neuro-enhancement technology. These results were obtained using a standard rTMS treatment protocol; therefore, the applicability of these findings to other stimulation protocols remains unknown.

## 6 Conclusion

In conclusion, this study demonstrates that rTMS can significantly improve proprioceptive task performance in ACLR patients, including enhanced balance control and joint position sense accuracy. However, the broader effects on motor function recovery and rehabilitation outcomes require further investigation.

## Data Availability

The raw data supporting the conclusions of this article will be made available by the authors, without undue reservation.
